# Clinical and Radiological Evolution of Idiopathic Normal Pressure Hydrocephalus: A Critical Review

**DOI:** 10.1002/mdc3.70419

**Published:** 2025-11-03

**Authors:** David Campo‐Caballero, Elissa Ash, Alfonso Fasano, Davide Martino, Mats Tullberg, Araceli Alonso Canovas, Joachim K. Krauss

**Affiliations:** ^1^ Department of Neurology, Movement Disorders Unit Hospital Universitario Donostia San Sebastián Spain; ^2^ Department of Neurosciences Biogipuzkoa Health Research Institute San Sebastián Spain; ^3^ Neurosciences University of the Basque Country (UPV/EHU) San Sebastian Spain; ^4^ Department of Neurology Tel Aviv Sourasky Medical Center Tel Aviv Israel; ^5^ Faculty of Medicine Tel Aviv University Tel Aviv Israel; ^6^ Department of Medicine, Division of Neurology University of Toronto Toronto Ontario Canada; ^7^ IRCCS Humanitas Research Hospital Milan Italy; ^8^ Department of Clinical Neurosciences and Hotchkiss Brain Institute University of Calgary Calgary Alberta Canada; ^9^ Hydrocephalus Research Unit, Department of Clinical Neuroscience, Institute of Neuroscience and Physiology, Sahlgrenska Academy University of Gothenburg Gothenburg Sweden; ^10^ Department of Neurology Sahlgrenska University Hospital Gothenburg Sweden; ^11^ Movement Disorders Unit, Department of Neurology Hospital Universitario Ramon y Cajal Madrid Spain; ^12^ Universidad de Alcala Madrid Spain; ^13^ Department of Neurosurgery Hannover Medical School Hannover Germany; ^14^ Center for Systems Neuroscience Hannover Germany

**Keywords:** cognitive impairment, critical review, gait disturbance, idiopathic normal pressure hydrocephalus, urinary dysfunction

## Abstract

**Background:**

The pathophysiology of idiopathic normal pressure hydrocephalus (iNPH) remains poorly understood. While it is commonly accepted that iNPH has an insidious onset, little is known about its preclinical and early stages and its development over time.

**Objectives:**

To gain more insight into how iNPH becomes manifest clinically and radiologically, and how its major clinical symptoms evolve in non‐shunted patients.

**Methods:**

For this critical review a literature search was performed using specific search terms concerning the evolution of iNPH. Manuscripts were categorized according to their content providing information on different domains including the early manifestation of clinical features, the evolution of the three major clinical symptoms, and the development of radiological findings.

**Results:**

Gait disturbance in general, is the earliest clinical symptom of iNPH. There is a gradual but variable decline within the first years resulting in a change of phenotype. Cognitive impairment varies widely depending on co‐morbidities. Urinary dysfunction evolves from urinary urgency to incontinence. Radiological features of iNPH such as ventricular enlargement, enlarged subarachnoid spaces, and flattening of sulci at the parasagittal high convexity are present in the preclinical stage of iNPH, but the sequence of their appearance remains unclear as well as the impact of white matter lesions.

**Conclusions:**

The evolution of iNPH shows remarkable heterogeneity. While there is a need to define distinct clinical stages, it is also important to better identify the preclinical stages of iNPH. Assessment of treatment outcomes needs to consider the stage of the disease at the time of intervention.

Idiopathic normal pressure hydrocephalus (iNPH) is a complex disorder characterized by disturbed cerebrospinal fluid (CSF) dynamics leading to ventricular enlargement and a variety of clinical symptoms including gait disturbance, cognitive impairment and urinary dysfunction.^1^ Although the clinical entity was described in the 1960s,^2^ its pathophysiology still remains poorly understood.^3^, ^4^ It is widely recognized that iNPH has an insidious clinical onset and a gradual clinical progression.^5^

Gait and balance disturbances have been identified as the most prominent clinical features of iNPH.^6^, ^7^ When cognitive impairment or urinary dysfunction occur in isolation or as the prominent clinical features, a critical revision of the diagnosis has been advised.^8^ It has further been suggested that the presence of the complete clinical triad may already correspond to a late phase of iNPH.^9^ Furthermore, it has been assumed that iNPH takes years to develop from a presymptomatic phase, and the initial features may be “soft and easy to miss.”^9^ However, there is very little data available on the preclinical and the very early stages of iNPH.

The evolution of radiological findings over time in asymptomatic individuals and the transition to symptomatic iNPH are poorly understood.^10^, ^11^, ^12^ Asymptomatic ventriculomegaly, enlarged subarachnoid spaces, and tight cortical sulci at the parasagittal convexity, which has also been termed *asymptomatic ventriculomegaly with iNPH‐like features on MRI (AVIM)*, may represent preclinical stages of the disease, with a subset of individuals progressing to develop clinical symptoms over periods of different temporal duration.^10^, ^11^, ^13^ Nevertheless, the factors determining the transition from preclinical stages to symptomatic iNPH are still largely unknown and possibly related to partially treatable risk factors such as sleep apnea or arterial hypertension.^14^, ^15^ Furthermore, the temporal sequence of the radiological changes and their association with white matter changes have not been investigated in detail in larger populations.^14^, ^16^

Finally, the course of iNPH progression appears to be not uniform, although most patients suffer deterioration of clinical symptoms within months or years.^3^ While it has been generally accepted that CSF shunting is the procedure of choice to treat iNPH, both patient selection for surgery and its timing remain a matter of debate.^3^, ^17^ The natural course of iNPH seems to be gradual, with worsening prognosis as the disease advances.^5^, ^18^ Therefore, establishing the optimal time for intervention in the early stages of the disease is warranted.

Here, we performed a critical review of the literature to gain more insight on how iNPH becomes manifest clinically and radiologically, and how clinical and radiological features evolve over time in non‐shunted patients, emphasizing the importance of identifying preclinical stages and incorporating imaging techniques early to enhance diagnostic accuracy and prognostic assessment.

## Methods

The study was conceived and conducted within the Normal Pressure Hydrocephalus Study Group of the International Parkinson and Movement Disorder Society.

For this critical review we performed a literature search of the PubMed databases using the terms “normal pressure hydrocephalus natural history,” “normal pressure hydrocephalus idiopathic development,” and “normal pressure hydrocephalus idiopathic evolution.” We selected original articles that referred to patients diagnosed with iNPH prior to CSF shunting, and which reported on the evolution and development of clinical features and radiological findings in accordance with PRISMA guidelines.^19^

Manuscripts were categorized according to whether they provided information on 1. the early manifestation of clinical symptoms, 2. the evolution of gait disturbance, 3. the evolution of cognitive impairment, 4. the evolution of urinary dysfunction, and 5. the evolution of radiological findings. Section‐specific terms were included in the search. Specifically, for the section on early manifestation of clinical features, we added the terms “clinical onset” and “first symptoms;” for the gait disturbance section, we used the terms “gait” and “gait dysfunction;” for the cognitive impairment section, we used the terms “cognition,” “cognitive dysfunction” and “dementia;” for the urinary dysfunction section, we used the terms “urinary incontinence,” “urinary urgency” and “urinary symptoms;” and for the radiological findings section, we also used the terms “ventriculomegaly,” “Evans’ index” and “disproportionately enlarged subarachnoid space hydrocephalus (DESH).”

**Figure 1:**
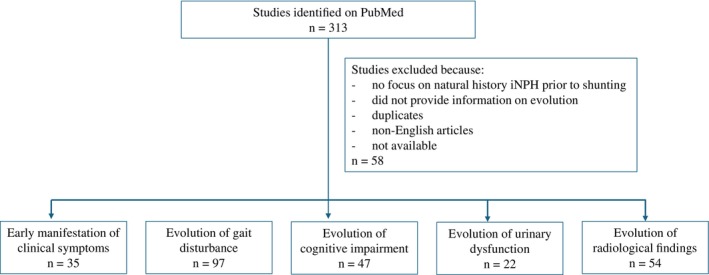
Summary of the critical review process to identify articles related to the evolution of idiopathic normal pressure hydrocephalus.

Articles written in a language other than English were excluded. The search start date corresponded to database inception. Full‐text articles that met the inclusion criteria were assessed for eligibility. A summary of the review process is demonstrated in Figure 1.

## Results

### Critical Review

The PubMed search performed in February 2025 yielded a total of 313 articles. Manuscripts were screened for relevance based on title and abstract, excluding those that did not focus on the natural history of iNPH prior to CSF shunting or did not provide information on clinical and radiological evolution. After exclusion of duplicates, articles that were not written in English, or that were not available for review, the number was reduced to 255 articles.

Manuscripts were then categorized according to providing information on 1. the early manifestation of clinical features (35 articles), 2. the evolution of gait disturbance (97 articles), 3. cognitive impairment (47 articles), 4. urinary dysfunction (22 articles), and 5. radiological findings (54 articles). Although all manuscripts contained some information on the evolution of iNPH, only few concentrated on such aspects and these articles form the basis for the following analysis.

### How Does iNPH Become Manifest Clinically?

Gait disturbance was the first clinical feature in all instances in a series of 10 patients who developed iNPH at a mean age of 77 years and who had cranial imaging studies more than 3 years before symptom onset.^12^ In a study including 429 patients with iNPH, gait disturbance was reported as the first symptom in 40% of patients, disturbed balance in 18%, memory impairment in 12%, incontinence in 7%, other symptoms in 3%, and a combination of symptoms had appeared in 18%.^6^

In a series of 132 patients with iNPH undergoing CSF shunting, gait disturbance (present in 130 [98%] patients) had been noted for an average of 36 ± 30 months before surgery, urinary dysfunction (present in 104 [79%] patients) for an average of 30 ± 28 months, and cognitive impairment (present in 103 [78%] patients) for an average of 30 ± 25 months,^20^ indicating that gait disturbance overall manifested earlier than the other features in this large group of patients.

In a longitudinal single‐case study, a patient who was ultimately diagnosed with iNPH and shunted had been previously followed with annual radiological and clinical examinations for 16 years prior to shunt surgery.^21^ This patient had signs of gait disturbance 7 years prior to diagnosis, followed by urinary dysfunction 1 year thereafter, and cognitive impairment 5 years later.

### Evolution of Gait Disturbance

The phenotype of the gait disturbance of iNPH has been labeled with various terms such as broad‐based gait, magnetic gait, shuffling gait, gait ataxia and others.^3^, ^22^ In a subset of patients, freezing of gait is also evident,^6^, ^23^ and postural instability has been noted in most patients.^1^, ^24^, ^25^ Gait analysis studies have provided several specific features of the gait disturbance of iNPH which are distinct but partially overlap those of Parkinson's disease.^26^, ^27^ An inherent problem of many published studies describing the gait disturbance in iNPH is that the severity of the gait disturbance has not been correlated with disease duration or with the severity of other features in individual patients.^27^ Thus, it remains difficult to outline details of the progression of the iNPH‐related gait disturbance.

Gait disturbance in isolation has been described as the first clinical manifestation in 73–98% of patients with a suspected diagnosis of iNPH, increasing up to 88–100% when present concomitantly with cognitive impairment.^12^, ^17^, ^20^

In a study on a cohort of 14 patients, worsening of gait was noted in 64% of patients after 3 to 4 months of observation.^28^ This is in line with a review summarizing the results of six studies reporting on the follow‐up of un‐shunted patients with iNPH, according to which the majority of patients had deteriorated at 3 months after the initial evaluation.^29^

The course of the progression of gait disturbance over a period of 12–24 months was described in a series of 9 patients with probable iNPH who refused shunt surgery.^30^ The gait disturbance had become worse in 33% of the patients after 12 months, while this was the case in 89% after 24 months. In a prospective study on a series of 51 patients (with 41 of them presenting with gait disturbance) who were referred for clinical evaluation of suspected iNPH, 26 patients were followed without having shunt surgery between 12 months and 5 years.^31^ One year after initial assessment, 63% had persistent or worsened gait disturbance, and after 5 years gait had worsened in 65%. It remained unclear, however, whether those patients without gait deterioration had indeed a diagnosis of iNPH.

A more comprehensive study on 33 patients with iNPH who had delayed shunt surgery (at least for 6 months) reported significant clinical deterioration between the first initial evaluation and a re‐evaluation just prior to surgery.^5^ There was an overall worsening of 23% on a specific clinical iNPH scale during a median of 13 months with a median deterioration of 3 points on a subscale for gait and of 17 points on a subscale for balance.

In a study which investigated the occurrence of freezing of gait in iNPH, disease duration of iNPH was significantly longer in patients with freezing (mean 4 years) than in those without (mean 2 years).^23^ Also, freezers had a slower walking speed (mean 0.57 m/s versus 0.87 m/s), and the Tinetti balance and gait scores (7 versus 11, and 3 versus 8, respectively) were significantly lower in these patients indicating that freezing of gait, in general, is a late‐stage feature of iNPH.

Regarding the clinical deterioration in untreated patients, it has been hypothesized that to maximize the benefits of treatment, surgery should be performed as soon as possible after diagnosis.^5^ In a prospective study, the average magnitude of post‐operative change at 3 months was similar between 33 patients with NPH who underwent surgery after a median of 13 months from diagnosis and 69 patients who were surgically treated within 3 months from diagnosis. However, since the delayed‐surgery group had clinically worsened more prior to shunting, their post‐operative outcome was still significantly poorer according to both the total score on the iNPH scale and the scores for gait and balance.^5^ The authors thus hypothesized that the deterioration which had occurred while waiting for surgery was only partially reversible. Moreover, survival was significantly worse in the group with delayed treatment for patients over the age of 75.^32^

In another study whose aim was to determine the efficacy of lumbo‐peritoneal shunt surgery in iNPH, 46 patients had surgery within 1 month after randomization while 41 patients had surgery postponed by 3 months.^33^ Changes in the primary outcome (modified Rankin Scale) and in secondary outcome measures (iNPH gait subscale, timed up‐and‐go test, walking test) differed significantly at 3 months after surgery with less improvement in the postponed surgery group, which however was not the case at 12 months follow‐up.

While in most patients with untreated iNPH a progressive deterioration of gait was reported, a small number of patients was described to have experienced spontaneous improvement, although the degree of improvement has not been well characterized.^29^, ^34^ It remains unclear whether such improvement was related to transient lumbar CSF drainage or to other factors such as physiotherapy or increased physical activity. For instance, in the above‐mentioned clinical trial on lumbo‐peritoneal shunting,^33^ two of 44 patients in the group in whom surgery was delayed for a median of 92 days had spontaneously improved during this longer pre‐operative period.

### Evolution of Cognitive Impairment

Cognitive decline in iNPH has been characterized mainly as a frontal‐subcortical dysfunction.^35^, ^36^, ^37^, ^38^, ^39^ The cognitive profile involves attentional deficits, psychomotor slowing, and memory decline.^40^ The memory deficits are less pervasive than those in Alzheimer's disease^39^ and have been described as resembling a subcortical memory dysfunction pattern more, where poor recall is aided by recognition cues.^40^ In addition, the development of apathy is common.^41^, ^42^ Hellström et al reported that neuropsychological deficits in iNPH patients were widely distributed, interrelated, associated with neurological signs, and aggravated by vascular comorbidity.^35^ A recent critical review of the literature showed that large observational studies are needed to screen cognition broadly using independently validated screening or assessment tools on sufficiently sized clinical samples and comparing patients with iNPH to age‐matched individuals from the general population.^1^

It has been suggested that cortical cognitive dysfunction is less apparent in iNPH as compared to other neurodegenerative diseases.^39^ Poorer cognition is associated with older age, longer disease duration, co‐morbidity, variable outcomes after shunt surgery, and increased mortality.^18^

Memory impairments have been described to appear as the first cognitive manifestation of iNPH.^43^, ^44^ Some studies have observed that visuospatial deficits, and not executive dysfunction, could be an early sign of cognitive deterioration in iNPH patients regardless of the severity of global cognitive dysfunction.^39^, ^45^

The evolution of cognitive symptoms in iNPH, however, has been examined in a limited fashion without definitive conclusions. Several studies demonstrated early executive dysfunction and psychomotor slowing,^40^ followed by more widespread cognitive decline at later stages.^46^ Yet, others reported also early and diffuse cognitive changes, including visuospatial dysfunction and memory impairments.^45^

A population‐based study followed up 53 patients with hydrocephalic ventricular enlargement and either probable iNPH (*n* = 24) or asymptomatic/possible iNPH (*n* = 29) who did not undergo shunting.^47^ The 5‐year mortality was 87.5% among those with probable iNPH. There was an increased risk of developing dementia in individuals with possible iNPH or asymptomatic hydrocephalic ventricular enlargement, of which 40% developed dementia compared to 21% of patients without ventricular enlargement.

In the above‐mentioned study by Andrén et al, who reported findings on 33 patients with iNPH who had delayed shunt surgery by at least 6 months,^18^ the significant overall clinical deterioration between the initial evaluation and re‐evaluation before surgery was accompanied by a decline from 25 to 22 on Mini Mental Score Examination (MMSE) testing. Remarkably, MMSE scores at the early post‐op assessment improved back to the initial score of 25, suggesting that the effect of surgical delay was not irreversible. Similar to gait and balance, the delayed surgery group had worse cognitive outcome measured on the iNPH scale compared to the early group.

A study with 104 community dwelling participants with radiological features compatible with iNPH who did not undergo surgery showed that, at a median follow‐up of 25 months, the neuropsychological trajectory for those developing iNPH included a reduction in declarative memory.^44^ In subjects with possible iNPH at baseline, the 2‐year follow‐up showed a worsening of executive dysfunction.

### Evolution of Urinary Dysfunction

Urinary dysfunction manifesting either as urge phenomena or as incontinence is the least studied clinical feature in iNPH. Overall, some type of urinary problems have been reported to manifest in 45 to 93% of patients with iNPH.^48^ A major obstacle to determine its iNPH‐related evolution is that urinary dysfunction is present in a large proportion of the general population older than 60 years, which may be secondary to various causes.^49^ Furthermore, there is a great disparity between the evaluation methodology of urinary dysfunction among different studies.^50^, ^51^

It has been suggested that patients may underreport their urinary disturbances due to cognitive impairment or become diaper‐bound due to gait dysfunction,^48^ and impairment caused by urinary dysfunction has been described to be much less relevant when compared to that from gait disturbances or memory impairment.^50^ In fact, no prospective or retrospective studies specifically regarding the natural evolution of urinary dysfunction in un‐shunted iNPH patients have been identified.

Overall, urinary incontinence has been regarded a late feature in the course of the disease^52^ preceded by urinary urgency, suggesting an early appearance of bladder overactivity in iNPH.^48^ In a study on 42 un‐shunted patients with possible iNPH, 93% had storage symptoms (small bladder capacity), 64% had urinary urgency/ increased frequency, and 57% had urinary incontinence.^48^ In a retrospective study on 81 patients, urinary urgency was found in 81% and incontinence in 70%.^51^ In another study on 55 patients, the most common symptom related to overactive bladder syndrome was nycturia followed by urgency, and lastly urgency incontinence.^50^ In summary, the findings of these studies indicate that urinary urgency/ increased frequency of micturition precedes urinary incontinence in iNPH.

### How Do Radiological Findings Evolve?

Over the decades it has been realized that in addition to ventriculomegaly, there are several other criteria which define the radiological picture of iNPH. The generally accepted radiological hallmarks of iNPH nowadays include ventricular hydrocephalus with an Evans index greater than 0.3, enlarged subarachnoid spaces concerning in particular the Sylvian fissures and focal dilatation of sulci (disproportionately enlarged subarachnoid space hydrocephalus, DESH), narrowing of the cortical sulci of the midline parasagittal convexity (tight high convexity, THC), and a callosal angle <90° measured at the splenium.^53^, ^54^, ^55^ MRI studies also demonstrate the presence of both periventricular and deep white matter lesions in a large proportion of iNPH patients which has been interpreted to indicate both the co‐existence of subcortical vascular encephalopathy in a subset of iNPH patients and a possible causal relationship towards the development of iNPH.^16^, ^56^, ^57^

While the dominant imaging studies for the radiological diagnosis of iNPH initially were pneumoencephalograpy or cisternography, these were replaced subsequently by computed tomography and thereafter by MRI. Notably, some of the radiological features which now are considered hallmarks of iNPH in addition to hydrocephalus such as a sharp callosal angle or DESH were described before the widespread distribution and routine use of MRI.^58^, ^59^ In fact, in the past decade it has been realized that both diagnostic accuracy and shunt responsiveness depend on considering such radiological features, which also help differentiate iNPH from neurodegenerative disorders including progressive supranuclear palsy and others.^17^ To increase the likelihood for establishing a radiological diagnosis of iNPH and to differentiate it from other disorders associated with hydrocephalus a radiological scale has been developed (the iNPH Radscale) that includes several imaging criteria.^60^

Attempts to define the presymptomatic imaging findings in iNPH face several difficulties. First, the clinical picture of iNPH may be syndromic and heterogenous rather than a uniform entity, associated with variable co‐pathologies and different pathophysiologic pathways and mechanisms, finally resulting in a convergent clinical picture.^4^ Furthermore, not all asymptomatic patients with iNPH‐like radiological features who were described in recent imaging studies were available for long‐term follow‐up, which may have contributed to the partial discrepancy of study findings.

The existence of a presymptomatic radiological state^61^ before the development of the clinical picture of iNPH was suggested more than 10 years ago.^62^ Some studies have indicated a prolonged process of several years, up to 10–15 years, before the clinical manifestation of iNPH.^12^

In an earlier study by Iseki et al,^10^ 8 asymptomatic patients with iNPH‐like features were identified upon MRI screening of a population of 1142 people older than 60 years. At follow‐up, 2 of these 8 had progressed to develop gait disturbance and dementia within a time span between 4 and 8 years. When the follow‐up was extended to 16 years, 5 of these 8 patients presented with clinical manifestations of iNPH.^11^ The same study found that the conversion to iNPH was more common if THC was present at baseline, vs. cases with only ventriculomegaly.^11^

Engel et al reported a series of 10 patients with iNPH in whom cranial imaging studies were available before the onset of symptoms.^12^ Remarkably, 9 already had typical imaging features of iNPH more than 3 years prior to any clinical symptom. Hydrocephalus with an Evans index >0.3 was present in all cases, but it had increased at the time when clinical symptoms became manifest. Seven of the 9 patients for whom appropriate images were available had THC which increased in 5 over time, but no major Sylvian fissure atrophy was described. In addition, all patients manifested white matter lesions. The authors concluded that the presence or absence of THC would instead be a dynamic process varying in a non‐linear fashion.

In contrast, Miyazaki et al^14^ proposed THC as the first MRI finding of iNPH at the preclinical stage. When they evaluated retrospectively a total of 2196 FDG‐PET CT studies in patients over 50 years of age, they found that 54 patients had DESH findings, most of them with THC even in the absence of hydrocephalus. However, given the retrospective nature of their study, it was difficult to establish a clear relationship with the development of clinical manifestations. In several patients with THC, there was gradual ventricular enlargement and slight enlargement of the Sylvian fissures on follow‐up. Notably, it has been repeatedly shown that DESH findings may occur without obvious ventriculomegaly. It remains unclear what proportion of these patients will develop iNPH later.^63^ It is also of interest that, in community‐based studies, DESH findings were related to worse cognitive function in comparison to people with normal MRI.^63^, ^64^ Another population‐based study reported that ventricular enlargement alone was associated with gait disturbance.^61^

Suehiro and colleagues compared MR images of 15 asymptomatic patients with iNPH‐like features to those of 45 patients with clinical expression of iNPH.^65^ At 1‐year MRI follow‐up, there was enlargement of both ventricular system and Sylvian fissures, alongside worsening of THC. While 6 of these 15 showed clinical expression at follow‐up, no clear predictors for clinical disease onset were identified. According to a study by Kimihira et al,^13^ the risk for progression from asymptomatic ventriculomegaly combined with DESH or THC to clinically evident iNPH is estimated at 17% per year.

There has been little investigation in the development of the morphology of ventricular enlargement in iNPH. A disproportionate parieto‐occipital and temporal ventricular dilatation has been described in one study suggesting that those patients are more prone to develop cognitive impairment.^66^

While it has been widely accepted that periventricular and deep white matter lesions are frequently present in iNPH,^16^, ^67^, ^68^ and vascular risk factors, in particular arterial hypertension, are considered possible risk factors for iNPH,^69^, ^70^ this field has also been under‐investigated, and it remains unclear when and how ventricular enlargement and DESH develop in these patients.^3^

## Conclusion

The natural evolution of iNPH is characterized by a gradual progression of clinical and radiological features. Understanding this evolution is essential for timely diagnosis and intervention. This review highlights the complexity of iNPH progression and underscores the need for enhanced diagnostic and therapeutic approaches.

Gait disturbance, often combined with impaired postural function, is the hallmark and, in general, the earliest clinical symptom of iNPH preceding other symptoms.^71^ Untreated patients experience progressive worsening of gait with a change in its phenotype, a variable course in different patients and freezing appearing as a late symptom. The gradual but variable decline in gait within the first few years after diagnosis, as outlined in several studies, suggests a window for early intervention. However, the variability in symptom progression and the potential for spontaneous, albeit rare, improvement indicate that individualized monitoring is crucial. Several studies suggest that delaying shunt surgery could lead to irreversible decline, not only concerning cognitive function, but also gait and balance, although few other studies indicate comparable improvement between early and delayed interventions.^5^, ^33^ Certainly, there is a need for further studies to determine the optimal timing of surgical treatment before symptoms may become irreversible and to optimize outcomes.

Cognitive impairment in iNPH appears to vary widely, with some patients exhibiting early and diffuse cognitive changes while others show more localized deficits initially.^37^, ^38^, ^39^, ^40^ The progression of cognitive symptoms in untreated patients is less predictable than that of gait disturbance, potentially due to the influence of other neurodegenerative processes and comorbidities. In this regard, it needs to be considered that some patients with Alzheimer's disease or other cognitive disorders might develop iNPH only after the onset of dementia. On the other hand, Alzheimer's pathology might result from a longstanding iNPH and the supposed role of the glymphatic impairment.^15^ Regardless, cognitive deterioration is a significant contributor to morbidity and poor outcome in iNPH. The lack of more detailed longitudinal data on cognitive evolution in un‐shunted patients highlights a critical gap in current research.

Urinary dysfunction, though less studied, is a common feature in iNPH. The evolution from urinary urgency to incontinence suggests an initial phase of bladder overactivity, progressing as the disease advances.^48^ Our review indicates that isolated urinary symptoms should prompt reconsideration of iNPH diagnosis, as such a constellation may be more indicative of other pathologies.^8^ The underreporting of urinary symptoms and variability in their assessment across studies, however, complicate our understanding of their progression and real impact.

Radiologically, iNPH exhibits a range of progressive changes, but at the present time, the interplay between asymptomatic ventriculomegaly, narrowing of the subarachnoid spaces, and white matter changes remains to be fully elucidated. The identification of THC possibly indicates a prodromal stage of iNPH, but a dynamic and non‐linear progression of radiological abnormalities may follow or not.^12^ The transition from asymptomatic ventriculomegaly to symptomatic iNPH also remains poorly understood, with a subset of individuals developing clinical symptoms over extended follow‐up.^11^

While there are numerous studies on the changes of clinical symptoms of iNPH after CSF shunting,^72^, ^73^, ^74^ there is very little data available on the effect of medical treatment or physical therapy on the course of iNPH. Although levodopa has been described to improve gait in patients with concomitant PD, it remains unclear whether this would have an effect on the further development of the disorder.^75^, ^76^ Further, a possible effect of cholinesterase inhibitors on the evolution of dementia in patients with concomitant Alzheimer's disease would deserve further attention. While physical therapy and exercise may have a positive effect on several measures of gait, the long‐term effects in unshunted patients need to be further elucidated.^77^, ^78^, ^79^, ^80^

A limitation of our study is that the literature search was conducted only on PubMed without considering other sources such as Cochrane, Embase and others. Further, our search did not include the terms “balance” and “postural instability.” When reviewing the phenotype of a disease, it is important to consider the potential bias introduced by clinical guidelines and required diagnostic criteria in published literature. For iNPH, two major guidelines exist. According to the International Guidelines, gait or balance disturbance is required for the diagnosis of iNPH.^38^ The Japanese Guidelines 3rd version requires at least one symptom concerning gait, cognitive or urinary function.^53^ It cannot be excluded that these specifications, although probably true for most patients, may lead to underreporting of cognitive impairment.

Overall, the evolution of iNPH shows remarkable heterogeneity in clinical presentation and progression, partially owing to the co‐occurrence with other disorders. Our review emphasizes the need for defining distinct stages of disease progression that incorporate both clinical and radiological features, also to better identify preclinical stages and predict disease progression. A suggested outline for such a definition is shown in Table 1. Assessing treatment outcome needs to consider the stage of the disease at the time of intervention.

**TABLE 1:** Schematic outline of a proposed model for the development (and evaluation) of idiopathic normal pressure hydrocephalus

Disease stage Measures, definitions	Radiological pre stage	Radiological iNPH, asymptomatic	iNPH early clinical	iNPH advanced clinical	iNPH very late clinical
Radiological changes	*Presence of any of the following findings*: Hydrocephalus Tight high convexity Enlarged subarachnoid spaces Callosal angle <90 degrees *Plus optional*: White matter lesions	*Combination of the following findings*: Hydrocephalus Tight high convexity Enlarged subarachnoid spaces Callosal angle <90 degrees *Plus optional*: White matter lesions	Radiological iNPH *Plus optional*: White matter lesions	Radiological iNPH *Plus optional*: White matter lesions	Radiological iNPH White matter lesions
Gait and/ or balance disturbance	‐	‐	Absent or mild, moderate or severe	Moderate	Severe
Cognitive impairment	‐	‐	Absent or mild or moderate	Mild, moderate or severe	Mild, moderate or severe
Urinary dysfunction	‐	‐	Absent or mild or moderate	Absent or mild or moderate	Mild, moderate or severe
Pathophysiology	Impairment of CSF dynamics	Impairment of CSF dynamics	Clinical iNPH with reversible signs	Clinical iNPH with reversible and possibly irreversible signs	Clinical iNPH with irreversible signs (neurodegeneration?)
Recommended intervention	Annual monitoring for clinical symptoms	Annual monitoring for clinical symptoms	Standardized assessment including tap test Repeated tap test or CSF shunt	Standardized assessment including tap test Hydrodynamic studies (?) CSF shunt	Standardized assessment including tap test Hydrodynamic studies (?) CSF shunt
Treatment effect (shunt)	Unknown (preventive?)	Unknown (preventive?)	Marked	Marked	Mixed
Necessary measures for clinical future research	Standardized quantification of radiological changes Check for co‐morbidity	Standardized quantification of radiological changes Check for co‐morbidity	Standardized quantification of clinical symptoms (e.g. tandem test, postural stability; executive function and attention; screening urinary function) Check for co‐morbidity	Standardized quantification of clinical symptoms (e.g. walking speed and stride length; executive function and attention; screening urinary function) Check for co‐morbidity	Standardized quantification of clinical symptoms (e.g. walking speed and stride length, freezing; executive function and attention; screening urinary function) Check for co‐morbidity

Furthermore, better understanding of the interplay between iNPH and coexisting conditions such as cerebrovascular disease will shed light on the mechanisms underlying the disorder and its variable progression. Future research should focus on longitudinal studies to clarify the natural history of iNPH, particularly in un‐shunted patients, to inform clinical decision‐making and improve outcomes.

## Author Roles

Research project: A. Conception, B. Organization, C. Execution.

Statistical Analysis: A. Design, B. Execution, C. Review and Critique.

Manuscript Preparation: A. Writing of the first draft, B. Review and Critique.

D.C.C.:1B, 1C, 2A, 2B, 3A, 3B

E.A.:1B, 1C, 2C, 3A, 3B

A.F.:2C, 3B

D.M.:2C, 3B

M.T.:2C, 3B

A.A.C.:1A, 1B, 2A, 2C, 3B

J.K.K.:1A, 1B, 1C, 2A, 2C, 3A, 3B

## Disclosures

**Ethical Compliance Statement:** We confirm that we have read the Journal's position on issues involved in ethical publication and affirm that this work is consistent with those guidelines. The study was conducted in accordance with the Declaration of Helsinki. For the present critical review no formal Ethical Commission approval and no informed consent was required.

**Funding Sources and Conflict of Interest:** No specific funding was received for this work. The authors declare that there are no conflicts of interest relevant to this work.

**Financial Disclosures for the previous 12 months:** DCC: Nothing to report. EA: Nothing to report. AF: Nothing to report. DM: Nothing to report. MT: Nothing to report. AAC: Nothing to report. JKK: Nothing to report. The authors declare that there are no additional disclosures to report.

## Data Availability

The data that support the findings of this study are available on request from the corresponding author.
